# Inhibition of cc chemokine receptor 10 ameliorates osteoarthritis via inhibition of the phosphoinositide-3-kinase/Akt/mammalian target of rapamycin pathway

**DOI:** 10.1186/s13018-024-04642-x

**Published:** 2024-03-01

**Authors:** Yan Luo, Feng Zhou, Xiaojing Wang, Runwei Yang, Yi Li, Xiaochun Wu, Bin Ye

**Affiliations:** 1 General Practice, Wuhan Puren Hospital, Wuhan, 430080 China; 2 Nutrition Department, Wuhan Puren Hospital, Wuhan, 430080 China; 3 Cardiology Department, Wuhan Puren Hospital, Wuhan, 430080 China; 4 Rheumatology Immunology Department, Wuhan Puren Hospital, Wuhan, 430080 China; 5 Orthopedics Department, Wuhan Huangpi People’s Hospital, Wuhan, 430300 China; 6 Orthopedics Department, Wuhan No. 9 Hospital, No. 20 Jilin Street, Wuhan, 430080 China

**Keywords:** CCR10, Osteoarthritis, PI3K/Akt/mTOR pathway

## Abstract

**Background:**

Osteoarthritis (OA) is a joint disease characterized by inflammation and progressive cartilage degradation. Chondrocyte apoptosis is the most common pathological feature of OA. Interleukin-1β (IL-1β), a major inflammatory cytokine that promotes cartilage degradation in OA, often stimulates primary human chondrocytes in vitro to establish an in vitro OA model. Moreover, IL-1β is involved in OA pathogenesis by stimulating the phosphoinositide-3-kinase (PI3K)/Akt and mitogen-activated protein kinases pathways. The G-protein-coupled receptor, cc chemokine receptor 10 (CCR10), plays a vital role in the occurrence and development of various malignant tumors. However, the mechanism underlying the role of CCR10 in the pathogenesis of OA remains unclear. We aimed to evaluate the protective effect of CCR10 on IL-1β-stimulated CHON-001 cells and elucidate the underlying mechanism.

**Methods:**

The CHON-001 cells were transfected with a control small interfering RNA (siRNA) or CCR10-siRNA for 24 h, and stimulated with 10 ng/mL IL-1β for 12 h to construct an OA model in vitro. The levels of CCR10, cleaved-caspase-3, MMP-3, MMP-13, Collagen II, Aggrecan, p-PI3K, PI3K, p-Akt, Akt, phosphorylated**-**mammalian target of rapamycin (p-mTOR), and mTOR were detected using quantitative reverse transcription polymerase chain reaction and western blotting. Viability, cytotoxicity, and apoptosis of CHON-001 cells were assessed using 3-(4,5-dimethylthiazol-2-yl)-2,5-diphenyltetrazolium bromide assay, lactate dehydrogenase assay (LDH), and flow cytometry analysis, respectively. Inflammatory cytokines (TNF-α, IL-6, and IL-8) were assessed using enzyme-linked immunosorbent assay.

**Results:**

Level of CCR10 was substantially higher in the IL-1β-stimulated CHON-001 cells than that in the control group, whereas CCR10 was down-regulated in the CCR10-siRNA transfected CHON-001 cells compared to that in the control-siRNA group. Notably, CCR10 inhibition alleviated IL-1β-induced inflammatory injury in the CHON-001 cells, as verified by enhanced cell viability, inhibited LDH release, reduced apoptotic cells, and cleaved-caspase-3 expression. Meanwhile, IL-1β induced the release of tumor necrosis factor alpha, IL-6, and IL-8, increase of MMP-3 and MMP-13, and decrease of Collagen II and Aggrecan in the CHON-001 cells, which were reversed by CCR10-siRNA. However, these effects were reversed upon PI3K agonist 740Y-P treatment. Further, IL-1β-induced PI3K/Akt/mTOR signaling pathway activation was inhibited by CCR10-siRNA, which was increased by 740Y-P treatment.

**Conclusion:**

Inhibition of CCR10 alleviates IL-1β-induced chondrocytes injury via PI3K/Akt/mTOR pathway inhibition, suggesting that CCR10 might be a promising target for novel OA therapeutic strategies.

## Background

Osteoarthritis (OA) is a chronic joint disease characterized by articular cartilage degeneration and secondary bone hyperplasia [1]. According to reports, the incidence of knee OA increases annually, placing a burden on families and healthcare systems. Recent studies have shown that inflammatory factors are involved in OA occurrence and development [2]. Among these cytokines, interleukin-1β (IL-1β) is considered the main inducer of OA, and it plays a key role in OA development. Xu et al. revealed that danshensu inhibits IL-1β-induced inflammatory response in chondrocytes and OA, possibly via suppression of the nuclear factor-κB (NF-κB) signaling pathway [3]. Moreover, Xiao et al. suggested that microRNA-613 alleviates IL-1β-induced injury in chondrogenic CHON-001 cells by targeting fibronectin 1 [4]. Furthermore, Jia et al. demonstrated that garcinol suppresses IL-1β-induced chondrocyte inflammation and OA via inhibition of the NF-κB signaling pathway [5]. Thus, inhibiting IL-1β-induced inflammatory response may be an effective strategy for the treatment of OA.

The chemokine receptor, G protein-coupled receptor, primarily located on the surface of white blood cells, is involved in the development of various cancers [6, 7]. The chemokine receptor, cc chemokine receptor 10 (CCR10), is expressed in various diseases [8, 9]. A previous study showed that CCR10/CCL27 crosstalk regulates cell metastasis via phosphoinositide-3-kinase (PI3K)-Akt signaling axis in non-small-cell lung cancer [10]. A recent study by Lin et al. suggested that CCR10 activation stimulates the invasion and migration of breast cancer cells through the extracellular signal-regulated kinase 1/2/matrix metalloproteinase-7 signaling pathway [11]. Additionally, CCR10 plays a vital role in monocyte migration in rheumatoid arthritis [12]. However, to date, no studies have confirmed the possible role of CCR10 in inflammation-driven OA.

Therefore, our study aimed to explain the roles of CCR10 in ameliorating the inflammatory response induced by IL-1β during OA development. We hypothesize that (i) the chondrocytes stimulated by IL-1β may induce chondrocyte injury, and was frequently applied for establishing the inflammation model in vitro; (ii) inhibition of CCR10 has a protective effect against OA in IL-1β-induced chondrocyte inflammation; and (iii) the underlying mechanisms of CCR10’s effects may be linked to the regulation of 740Y-P and PI3K/Akt/mTOR signaling pathway. Our findings indicated that CCR10 is a promising therapeutic agent for OA.

## Methods

### Cell culture and establishment of OA model

The CHON-001 cells were purchased from ATCC and maintained in Dulbecco’s Modified Eagle medium supplemented with 10% fetal bovine serum (Procell) and 1% penicillin/streptomycin (Procell) in an incubator containing 5% CO_2_ at 37˚C. An OA model for CHON-001 cells was established. Briefly, cells were transferred to the cultural condition with IL-1β (10 ng/mL) and cultured for 12 h. After incubation, the cells were acquired for subsequent experiments.

### Western blot assay

The CHON-001 cells were seeded in 96-well plates. The cells were then lysed using radioimmunoprecipitation assay buffer for 30 min on ice. Proteins were resolved using sodium dodecyl sulfate-polyacrylamide gel electrophoresis and transferred onto polyvinylidene difluoride membranes. The membranes were blocked with 5% skimmed milk for 2 h and then cultivated with primary antibodies against CCR10, cleaved-caspase-3, MMP-3. MMP-13, Collagen II, Aggrecan, p-PI3K, PI3K, p-Akt, Akt, p-mTOR, mTOR, GAPDH, or β-actin (1:1000, ASPEN) at 4 °C overnight. After washing thrice with Tris-buffered saline with 0.1% Tween® 20 detergent, the membranes were incubated with secondary antibodies for 2 h. Protein signals were visualized using enhanced chemiluminescence detection reagents and quantified using ImageJ software.

### Quantitative reverse transcription polymerase chain reaction (qRT-PCR) analysis

After treatment, the levels of CCR10 were measured using qRT-PCR. We isolated RNA from the CHON-001 cells using TRIpure total RNA extraction reagent (ELK Biotechnology), according to the manufacturer’s protocol. Total RNA was reverse transcribed to cDNA using a cDNA synthesis kit (ELK Biotechnology), and the qRT-PCR analysis was conducted using the SYBR Green Master mix with an ABI 7500 Real-Time PCR System (Life Technologies). Target gene expressions were performed using 2^−ΔΔCt^ method.

### Cell transfection

After the treatment, the CHON-001 cells were transfected with control-siRNA, CCR10-siRNA using Lipofectamine® 3000 reagent (Thermo) for 24 h, according to the instructions. The qRT-PCR and western blotting were performed to evaluate cell transfection efficiency.

### 3-(4,5-Dimethylthiazol-2-yl)-2,5-diphenyltetrazolium bromide (MTT) assay

After the treatment, the CHON-001 cells were implanted into 96-well plates and cultured for 24 h at 37 °C. The cells were treated with 10 µL of MTT solution and continuously incubated for further 4 h. Afterwards, the solution was removed and 100 µL of dimethyl sulfoxide was added to each well to solubilize the formazan product. Finally, the optical density at 570 nm was measured using a microplate reader (BioTek, Richmond, USA) after 15 min of vibration mixing, according to the manufacturer’s instructions.

### Lactate dehydrogenase (LDH) assay

Lactate dehydrogenase assay was used to determine CHON-001 cell viability. Briefly, the CHON-001 cells were cultured in 96-well plates. After the treatment, the cell culture medium in each well was removed, and LDH activity was determined using an LDH assay kit (Beyotime), according to the manufacturer’s instructions. Absorbance was measured at 450 nm using a microplate reader (BioTek, USA).

### Flow cytometry assay

After the treatment, the CHON-001 cells were cultured in 96-well plates. The cells were then collected by centrifugation at 4 °C for 5 min. The cells were then washed twice with phosphate buffered saline and assessed using the Annexin V/propidium iodide apoptosis detection kit (Beyotime). The cells were gently mixed and incubated for 20 min at room temperature in the dark. Finally, the apoptotic cells were analyzed using a flow cytometer (Beckman Coulter, Brea, CA, USA) and Kaluza, according to the manufacturer’s instructions.

### Enzyme-linked immunosorbent assay (ELISA) assay

After the treatment, the CHON-001 cells were collected and centrifuged for 10 min at 400 × g. The inflammatory cytokines (TNF-α, IL-6, and IL-8) in the CHON-001 cells were then quantified using ELISA kits (BioLegend, USA), according to the instructions. The value of the optical density at 450 nm was determined using a Multiscan Spectrum (Bio-Tek, China), according to the manufacturer’s instructions.

### Statistical analysis

Statistical analyses were conducted using SPSS 20.0. All the results are expressed as mean ± SD from three independent experiments. Differences among the groups were estimated using the unpaired Student’s t-test or one-way analysis of variance. Statistical significance was set at *P* < 0.05.

## Results

### CCR10 was up-regulated in IL-1β-induced CHON-001 cells

**Fig. 1:**
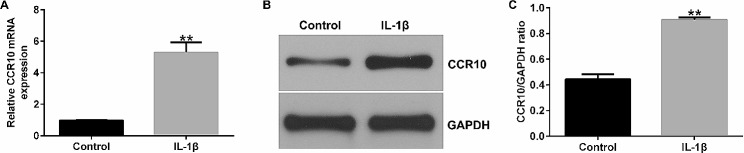
Induction of CCR10 expression in CHON-001 cells by IL-1β. CHON-001 cells were exposed to 10 ng/mL of IL-1β for 12 h. (**A**-**B**) Levels of CCR10 in control and IL-1β groups was determined by qRT-PCR and Western blot assay. (**C**) Ratio of CCR10 and GAPDH. **P < 0.01

First, 10 ng/mL of IL-1β was applied to stimulate the CHON-001 cells for 12 h to establish inflammatory damage of chondrocytes. After that, the levels of CCR10 in the CHON-001 cells were determined using qRT-PCR and western blotting. As displayed in Fig. 1A-C, the level of CCR10 was remarkably higher in the IL-1β-stimulated chondrocyte than that in the control group. Our findings revealed that IL-1β induced CHON-001 cell injury in OA.

### CCR10-siRNA inhibited CCR10 levels in IL-1β-treated CHON-001 cells

**Fig. 2:**
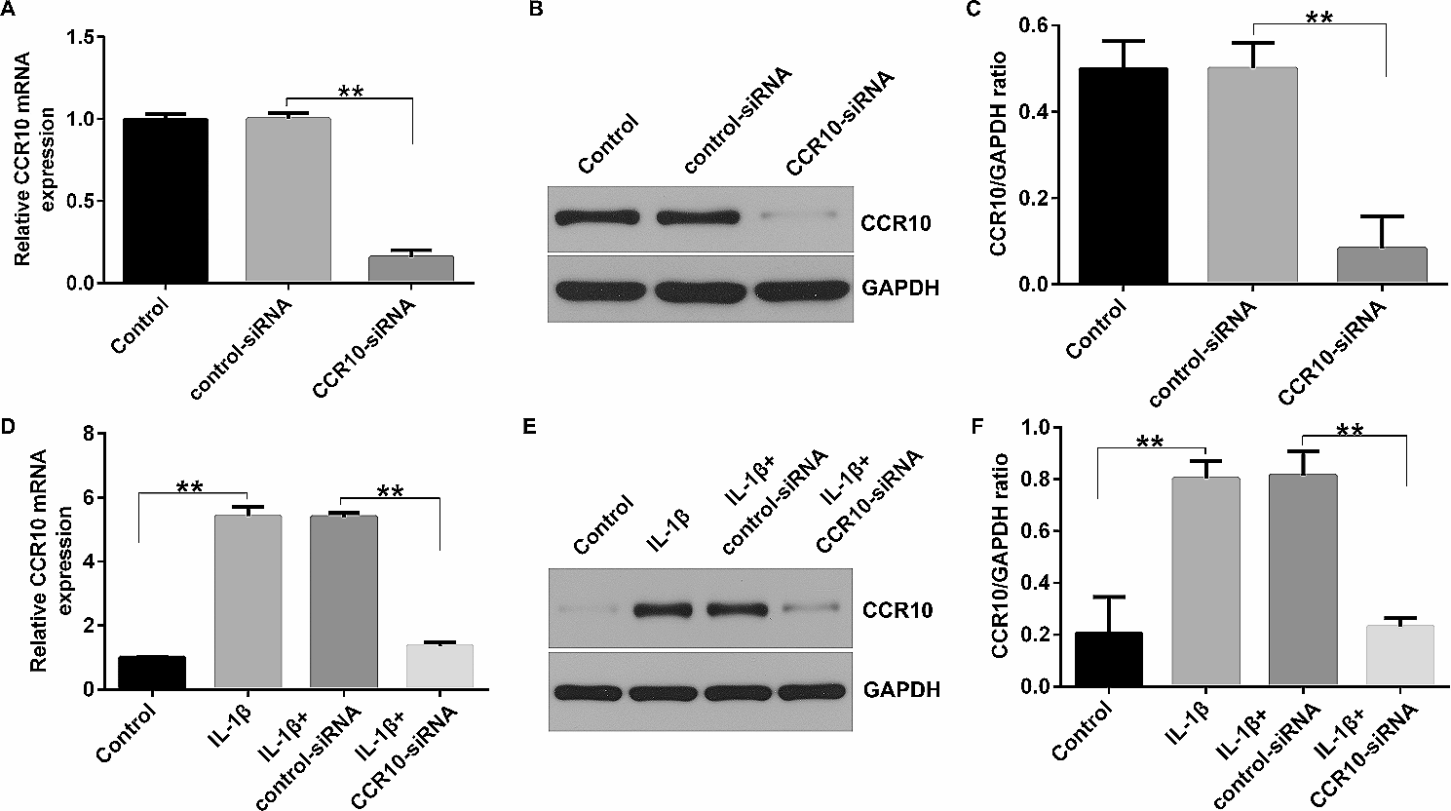
Reversal of the effects of IL-1β on CCR10 expression by CCR10-siRNA in CHON-001 cells. (**A**-**C**) Levels of CCR10 in control-siRNA or CCR10-siRNA group was assessed by qRT-PCR analysis and Western blot assay. (**D**) qRT-PCR analysis of CCR10 mRNA levels in control, IL-1β, IL-1β + control-siRNA, or IL-1β + CCR10-siRNA group. (**E** and **F**) Detection of CCR10 expression in the control, IL-1β, IL-1β + control-siRNA, or IL-1β + CCR10-siRNA group using a Western bolt assay. **P < 0.01

To further reveal the roles of CCR10 in OA, the CHON-001 cells were transfected with control-siRNA or CCR10-siRNA for 24 h, and induced by 10 ng/mL IL-1β for another 12 h. Results from Fig. 2A-C suggested that the level of CCR10 was lower in the CCR10-siRNA transfected cells compared to that in the control-siRNA group. Moreover, our observations demonstrated that 10 ng/mL of IL-1β remarkably enhanced CCR10 expression in the CHON-001 cells, while this promotion was inhibited after the CCR10-siRNA treatment (Fig. 2D-F). Based on these findings, we conclude that CCR10 is involved in OA progression.

### CCR10 silence alleviated IL-1β-stimulated chondrocyte inflammatory injury in OA

**Fig. 3:**
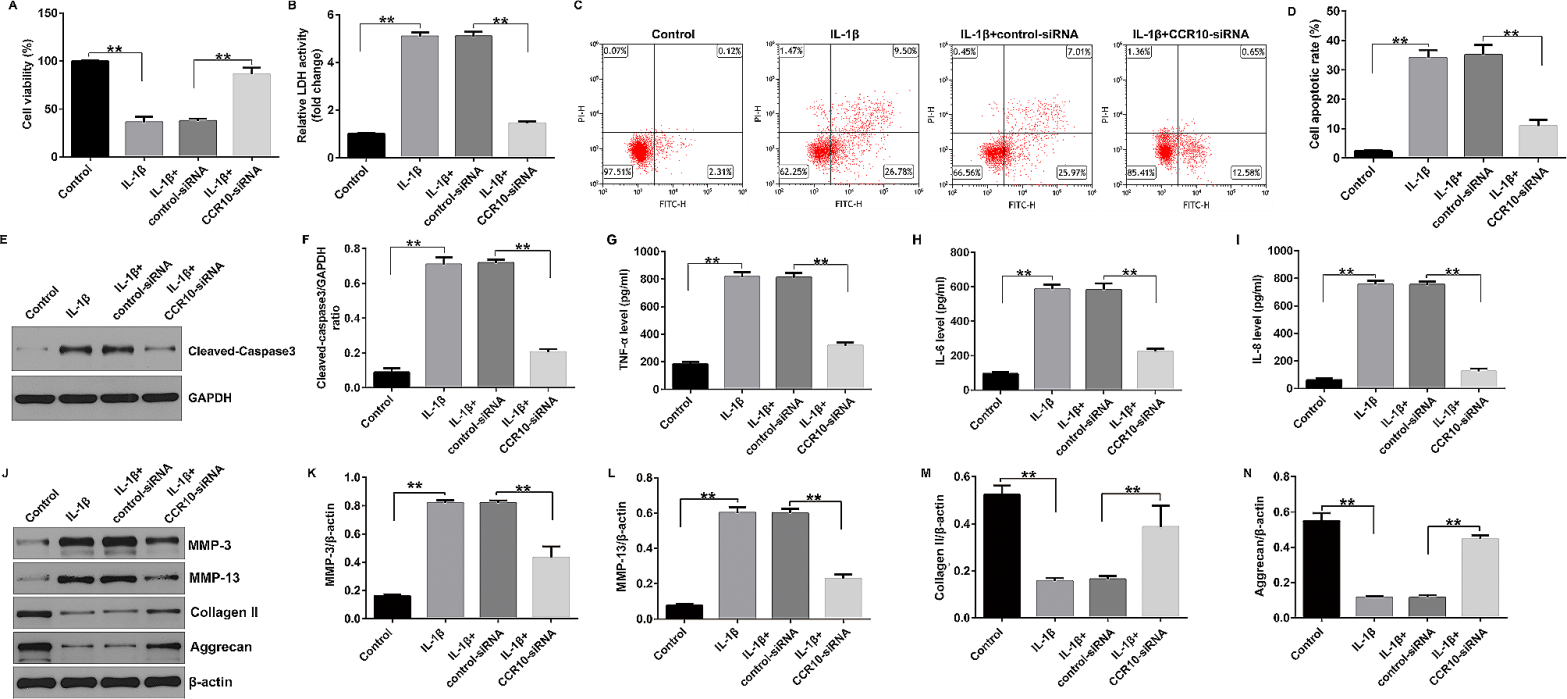
Effects of CCR10-siRNA on IL-1β-induced cell viability, apoptosis and inflammatory cytokines secretion. The CHON-001 cells were divided into four groups: control, IL-1β, IL-1β + control-siRNA, or IL-1β + CCR10-siRNA group. (**A**) Cell viability was assessed using MTT assay. (**B**) Analysis of LDH release. (**C**) Apoptosis was assessed by flow cytometry. (**D**) Quantification of apoptotic CHON-001 cells. (**E**) Western blot analysis of cleaved-caspase-3 expression. (**F**) Relatively cleaved-caspase-3 protein expression were quantified. The secretion of TNF-α (**G**), IL-6 (**H**) and IL-8 (**I**) were evaluated by ELISA. (**J**) Western blot analysis of MMP-3, MMP-13, Collagen II, and Aggrecan. (**K**) MMP-3/β-actin ratio. (**L**) MMP-13/β-actin ratio. (**M**) Collagen II /β-actin ratio. (**N**) Aggrecan/β-actin ratio. **P < 0.01

Having illustrated the relationship between CCR10 and OA, we investigated the biological functions of the CHON-001 cells after CCR10-siRNA treatment. We found that IL-1β led to inhibited cell viability (Fig. 3A), promoted LDH release (Fig. 3B), enhanced cells apoptosis (Fig. 3C and D), and enhanced cleaved-caspase-3 expression (Fig. 3E and F), while we found the opposite data in the CCR10-siRNA treated cells, suggesting that down-regulation of CCR10 relieved IL-1β-stimulated CHON-001 cells viability and apoptosis. Moreover, we explained the effects of CCR10-siRNA on inflammatory release in chondrocytes, including TNF-α, IL-6, and IL-8. The ELISA assay revealed that the secretion of inflammatory elements were obviously enhanced in the IL-1β-induced cells, and CCR10-siRNA inhibited inflammatory response, as opposed to the IL-1β + control-siRNA group (Fig. 3G-I). In addition, CCR10-siRNA significantly reduced MMP-3 and MMP-13 expression, and enhanced Collagen II and Aggrecan expression in IL-1β-induced CHON-001 cells, as opposed to the IL-1β + control-siRNA group (Fig. 3J-N). Our results revealed that CCR10 silence remitted IL-1β-induced cell viability, apoptosis, and inflammatory response in the CHON-001 cells.

### CCR10 silence inhibited IL-1β-induced chondrocyte injury by regulating the PI3K/Akt/mTOR pathway

**Fig. 4:**
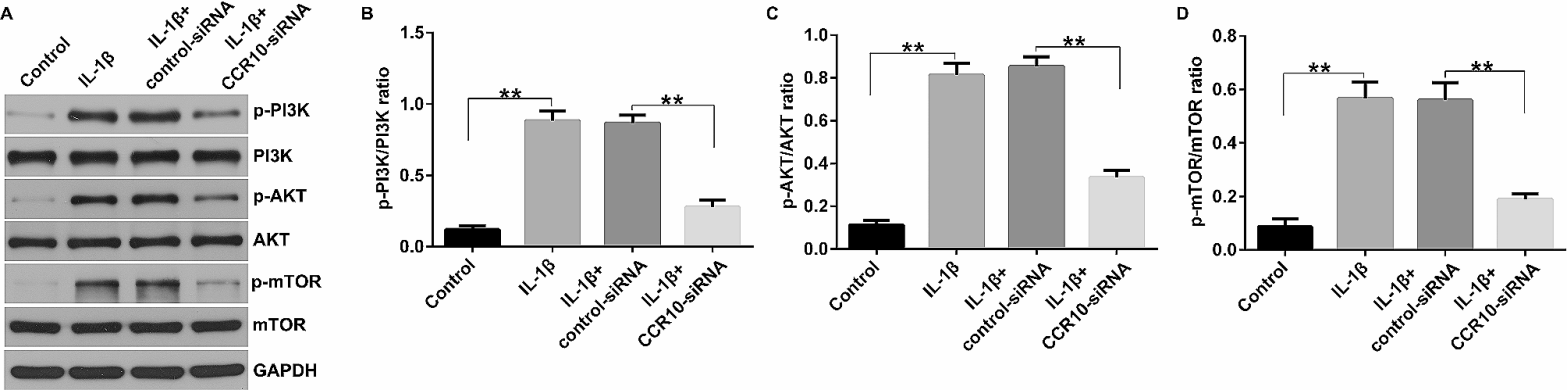
Effects of CCR10-siRNA on the PI3K/Akt/mTOR pathway in CHON-001 cells. The CHON-001 cells were divided into four groups: Control, IL-1β, IL-1β + control-siRNA, or IL-1β + CCR10-siRNA group. (**A**) The expression levels of p-PI3K, p-Akt, and p-mTOR were determined using Western blotting. (**B**-**D**) The p-PI3K/PI3K, p-Akt/Akt, and p-mTOR/mTOR ratios were quantified. **P < 0.01

A growing number of studies have demonstrated that the PI3K/Akt/mTOR pathway plays a vital role in inflammatory responses in many diseases [13]. Therefore, we investigated the role of CCR10 in the PI3K/Akt/mTOR pathway. The CHON-001 cells were transfected with the control-siRNA or CCR10-siRNA for 24 h, and stimulated with 10 ng/mL of IL-1β for another 12 h. Western blot assay was applied for determining the expression of p-PI3K, PI3K, p-Akt, Akt, p-mTOR, and mTOR in the PI3K/Akt/mTOR pathway. As shown in Fig. 4A, upregulation of p-PI3K, p-Akt, and p-mTOR was observed in the IL-1β-induced CHON-001 cells compared to that in the control group. However, we observed the opposite results in the CCR10-siRNA treated cells. Besides, the ratio of p-PI3K/PI3K (Fig. 4B), p-AKT/AKT (Fig. 4C), and p-mTOR/mTOR (Fig. 4D) were enhanced in IL-1β-induced CHON-001 cells, and markedly suppressed in the IL-1β + CCR10-siRNA group. Taken these data together, CCR10 inhibition alleviated IL-1β-stimulated human chondrocytes injury through the PI3K/Akt/mTOR pathway.

### 740Y-P reversed the effects of CCR10-siRNA on the PI3K/Akt/mTOR signaling pathway

**Fig. 5:**
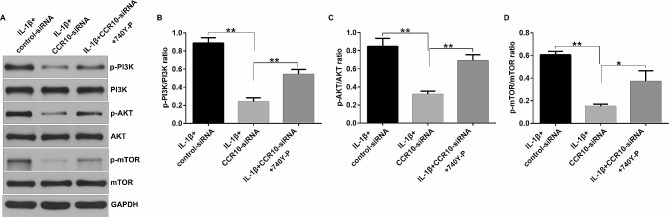
Effects of CCR10-siRNA or 740Y-P on PI3K/Akt/mTOR pathway. The CHON-001 cells were divided into three groups: IL-1β + control-siRNA, IL-1β + CCR10-siRNA, and IL-1β + CCR10-siRNA + 740Y-P group. (**A**) p-PI3K, p-Akt, and p-mTOR levels were determined using Western blotting. (**B**-**D**) Quantification of the p-PI3K/PI3K, p-Akt/Akt, and p-mTOR/mTOR ratios. * *P* < 0.05, **P < 0.01

This study aimed to explore the detailed mechanism of action of CCR10-siRNA on OA, and the PI3K agonist 740Y-P was used. The CHON-001 cells were treated with or without 20 µM 740Y-P/control-siRNA/CCR10-siRNA, and 10 ng/mL IL-1β for 12 h. Results from the western blot assay demonstrated that 740Y-P reversed the effects of CCR10-siRNA on the PI3K/Akt/mTOR signaling pathway, as verified by enhanced p-PI3K, p-AKT, and p-mTOR expression (Fig. 5A), as well as increased p-PI3K/PI3K, p-Akt/Akt, and p-mTOR/mTOR ratio (Fig. 5B-D). All these findings revealed that CCR10 inhibition alleviated IL-1β-stimulated human chondrocyte injury through the PI3K/Akt/mTOR pathway.

### 740Y-P reversed the effects of CCR10-siRNA on IL-1β-stimulated CHON-001 cell viability, apoptosis, and inflammatory response

**Fig. 6:**
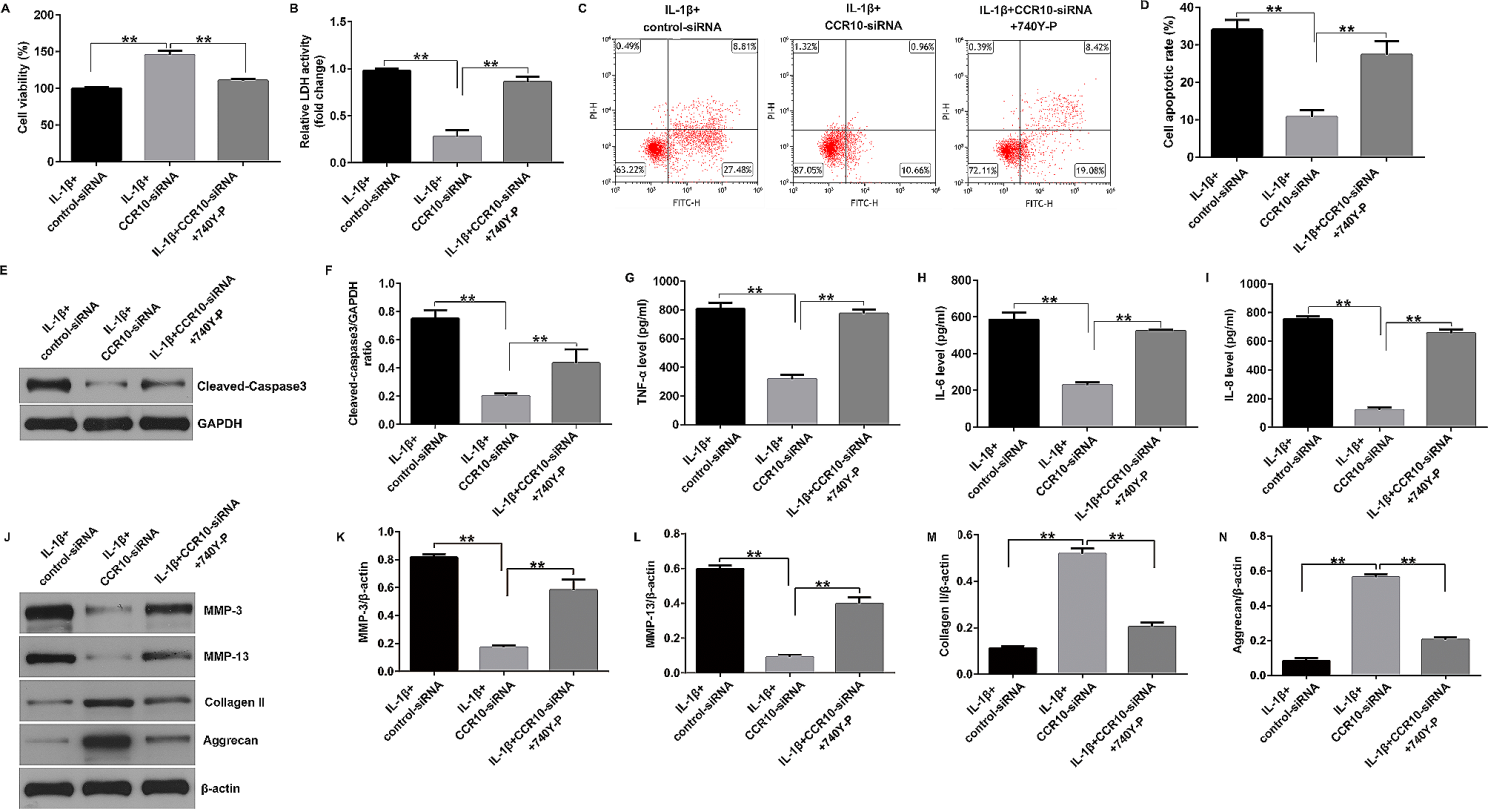
Effects of CCR10-siRNA or 740Y-P on IL-1β-induced cell viability, apoptosis, and inflammatory cytokines secretion. The CHON-001 cells were divided into three groups: IL-1β + control-siRNA, IL-1β + CCR10-siRNA, and IL-1β + CCR10-siRNA + 740Y-P groups. (**A**) MTT assay for cell viability. (**B**) Determination of LDH level. (**C**) Apoptotic cells were evaluated using flow cytometry. (**D**) Quantification of the apoptotic cells. (**E**) Determination of cleaved-caspase-3 expression. (**F**) Quantization of cleaved-caspase-3 expression. The levels of TNF-α (**G**), IL-6 (**H**), and IL-8 (**I**) were analyzed using ELISA. (**J**) Western blot analysis of MMP-3, MMP-13, Collagen II, and Aggrecan. (**K**) MMP-3/β-actin ratio. (**L**) MMP-13/β-actin ratio. (**M**) Collagen II /β-actin ratio. (**N**) Aggrecan/β-actin ratio. **P < 0.01

Moreover, we assessed the effects of 740Y-P on the biological functions of IL-1β-stimulated CHON-001 cells, including cell viability, apoptosis, and inflammatory response. Our results suggested that CCR10-siRNA notably increased the growth of IL-1β-stimulated CHON-001 cells (Fig. 6A), decreased LDH release (Fig. 6B), reduced cell apoptosis (Fig. 6C and D), and reduced cleaved-caspase-3 expression (Fig. 6E and F), as opposed to IL-1β + control-siRNA group. Besides, the silence of CCR10 reduced the secretion of inflammatory factors (TNF-α, IL-6, and IL-8) (Fig. 6G-I). Moreover, CCR10-siRNA significantly reduced MMP-3 and MMP-13 expression, and enhanced Collagen II and Aggrecan expression in IL-1β-induced CHON-001 cells, as opposed to the IL-1β + control-siRNA group (Fig. 6J-N). However, all these effects were successfully reversed by 740Y-P. Therefore, our data demonstrated that CCR10 inhibition alleviated injury in IL-1β-induced human chondrocytes by targeting 740Y-P through the PI3K/Akt/mTOR pathway.

## Discussion

Osteoarthritis is an inflammatory disease characterized by joint dysfunction, degeneration, and destruction of articular cartilage. Previous studies have shown that various factors, including strain, trauma, and joint deformity, lead to OA [14, 15]. Approximately three million new cases of OA occur annually. The incidence of OA increases annually with an increase in the population age [16]. The current treatment strategies for OA include physical therapy and analgesics [17]. However, these treatments provide only symptomatic relief. Therefore, there is an urgent need for effective drugs that inhibit the occurrence and progression of OA. Various investigations have suggested that IL-1β is a main inducer of OA. Xiao et al. suggested that microRNA-613 alleviates IL-1β-induced chondrogenic CHON-001 cell injury by targeting fibronectin 1 [4]. Yang et al. revealed that down-regulation of microRNA-23b-3p alleviates IL-1β-induced injury in chondrogenic CHON-001 cells [18]. In our study, the CHON-001 cells were stimulated with 10 ng/mL of IL-1β for 12 h to establish an OA model in vitro.

A chemokine receptor in humans, CCR10, is normally expressed by melanocytes, plasma cells, and skin-homing T-cells. Accumulating evidence suggests that CCR10 is involved in cancer progression. Kunkel et al. suggested that CCR10 expression is a common feature of circulating and mucosal epithelial tissue IgA antibody-secreting cells [19]. Furthermore, Wu et al. revealed that CCR10 promotes inflammation-driven hepatocarcinogenesis through PI3K/Akt pathway activation [20]. Based on these findings, our study aimed to evaluate the protective effect of CCR10 on IL-1β-stimulated CHON-001 cells, and we found that CCR10 was up-regulated in IL-1β-induced CHON-001 cells compared to that in the control group, which were in line with other studies, demonstrating that CCR10 potentially contributes to the development of OA.

Multiple studies have shown that abnormal CCR10 expression is instrumental in disease treatment. Simonetti et al. revealed a potential role of CCR10 expression in melanoma progression and immune escape [21]. Thus, in this study, control-siRNA or CCR10-siRNA was transfected into CHON-001 cells. We observed that CCR10-siRNA inhibited CCR10 levels in IL-1β-treated CHON-001 cells. Moreover, Li et al. suggested that the CCL27-CCR10 axis contributes to promoting lung squamous cell carcinoma proliferation, migration, and invasion [22]. Moreover, Chen et al. demonstrated that CCR10 upregulation is essential for glioma proliferation, invasion, and patient survival [23]. We evaluated the effects of CCR10-siRNA on the biological functions of CHON-001 cells, including cell proliferation, LDH levels, and apoptosis. Our data indicated that IL-1β led to reduced CHON-001 cells viability, promoted LDH release, increased apoptotic cells, and enhanced cleaved-caspase-3 expression. However, these findings were reversed in CCR10-siRNA transfected cells, suggesting that CCR10 contributes to OA pathogenesis by regulating CHON-001 cell proliferation and apoptosis.

It is well known that inflammation is a vital regulator in OA progression. In the pathological process of OA, inflammatory mediators, especially IL-1β, promote the secretion of other proinflammatory cytokines, thereby promoting the catabolic response of chondrocytes and damaging the structure of joint cartilage [24, 25]. Thus, inhibition of inflammation is considered a strategy to delay OA progression. We then determined the release of inflammatory factors in IL-1β-induced CHON-001 cells, including TNF-α, IL-6, and IL-8. The ELISA results revealed that IL-1β obviously stimulated the release of inflammatory factors, as compared to that in the control group. However, we observed the opposite results in the IL-1β + CCR10-siRNA group. Cartilage-related proteins such as Collagen II and Aggrecan were also determined in this study. The data indicated that IL-1β significantly reduced Collagen II and Aggrecan protein expression in CHON-001 cells, while this reduction was significantly reversed by CCR10-siRNA. Therefore, inhibition of chondrocyte apoptosis and inflammatory response may be beneficial for OA therapy. Signaling molecule associated with activated chemokine receptor include PI3K/Akt, NF-κB, and MAPK. A study has indicated that the PI3K/Akt signaling pathway is upregulated in CCL27 and CCR10 cells and related to cell proliferation, migration, and survival [23]. Xu et al. suggested that sirtuin 3 ameliorates OA by regulating chondrocyte autophagy and apoptosis via the PI3K/Akt/mTOR pathway [26]. However, few investigations have been conducted on the downstream signaling pathway mediated by CCR10 in OA. Therefore, we investigated the role of CCR10 in the PI3K/Akt/mTOR pathway. Our data revealed that CCR10 silence reversed the effects of IL-1β on the PI3K/Akt/mTOR pathway in CHON-001 cells, as confirmed by reduced p-PI3K, p-Akt, and p-mTOR expression, as well as suppressed p-PI3K/PI3K, p-Akt/Akt, and p-mTOR/mTOR ratio. These results indicated that CCR10 inhibition alleviated IL-1β-stimulated chondrocyte injury via the PI3K/Akt/mTOR pathway.

Finally, we explored whether CCR10 affects IL-1β-stimulated CHON-001 cells by directly regulating the PI3K/AKT pathway, and the PI3K agonist 740Y-P was used. We observed enhanced p-PI3K, p-Akt, and p-mTOR expression, as well as increased p-PI3K/PI3K, p-Akt/Akt and p-mTOR/mTOR ratio in IL-1β + CCR10-siRNA + 740Y-P-treated cells compared to the IL-1β + CCR10-siRNA-treated group. Further functional assays suggested that 740Y-P reversed the effects of CCR10-siRNA on IL-1β-stimulated CHON-001 cell viability and apoptosis, as shown by the reduced cell viability, enhanced LDH release, promoted apoptotic cells, and cleaved-caspase-3 expression. Moreover, in this study, 740Y-P notably enhanced the secretion of IL-6, IL-8, and TNF-α, increased MMP-3 and MMP-13 expression, and reduced Collagen II and Aggrecan protein expression in the IL-1β + CCR10-siRNA treated chondrocyte cells. Our findings demonstrated that CCR10 inhibition alleviated IL-1β-induced chondrocyte injury by targeting the PI3K/Akt/mTOR pathway.

Based on the above investigations, down-regulation of CCR10 relieved OA progression through inhibition of inflammatory response induced by IL-1β through PI3K/Akt/mTOR pathway regulation. Therefore, CCR10 may be a promising therapeutic target for OA. Additional in vivo experiments are required to elucidate the precise mechanism of action of CCR10 in OA development.

## Conclusions

Down-regulation of CCR10 relieved IL-1β induced chondrocyte cells injury through inhibition of inflammatory response and cell apoptosis through PI3K/Akt/mTOR pathway regulation.

## Data Availability

The datasets used and/or analyzed during the current study are available from the corresponding author on reasonable request.
