# Correction: Inhibition of cc chemokine receptor 10 ameliorates osteoarthritis via inhibition of the phosphoinositide-3-kinase/Akt/mammalian target of rapamycin pathway

**DOI:** 10.1186/s13018-025-05807-y

**Published:** 2025-05-14

**Authors:** Yan Luo, Feng Zhou, Xiaojing Wang, Runwei Yang, Yi Li, Xiaochun Wu, Bin Ye

**Affiliations:** 1 General Practice, Wuhan Puren Hospital, Wuhan, 430080 China; 2 Nutrition Department, Wuhan Puren Hospital, Wuhan, 430080 China; 3 Cardiology Department, Wuhan Puren Hospital, Wuhan, 430080 China; 4 Rheumatology Immunology Department, Wuhan Puren Hospital, Wuhan, 430080 China; 5 Orthopedics Department, Wuhan Huangpi People’s Hospital, Wuhan, 430300 China; 6 Orthopedics Department, Wuhan No. 9 Hospital, No. 20 Jilin Street, Wuhan, 430080 China

**Correction to: Journal of Orthopaedic Surgery and Research (2024) 19:158 ** 10.1186/s13018-024-04642-x

In this article Figs. 3 and 6 appeared incorrectly and have now been corrected in the original publication. For completeness and transparency, the old incorrect versions are displayed below.

Incorrect Fig. 3:

**Fig. 3:**
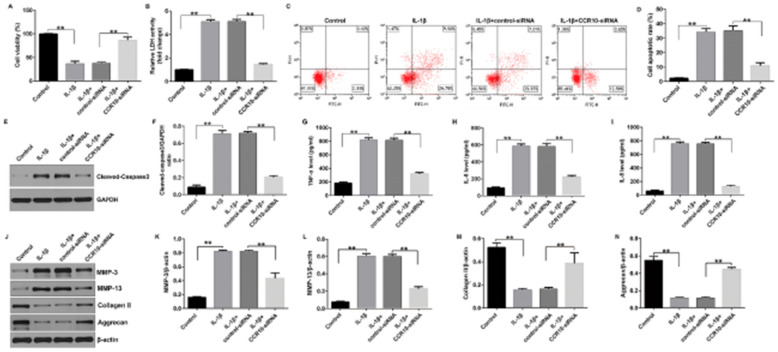
Effects of CCR10-siRNA on IL-1β-induced cell viability, apoptosis and inflammatory cytokines secretion. The CHON-001 cells were divided into four groups: control, IL-1β, IL-1β + control-siRNA, or IL-1β + CCR10-siRNA group. (**A**) Cell viability was assessed using MTT assay. (**B**) Analysis of LDH release. (**C**) Apoptosis was assessed by flow cytometry. (**D**) Quantification of apoptotic CHON-001 cells. (**E**) Western blot analysis of cleaved-caspase-3 expression. (**F**) Relatively cleaved-caspase-3 protein expression were quantified. The secretion of TNF-α (**G**), IL-6 (H) and IL-8 (**I**) were evaluated by ELISA. (**J**) Western blot analysis of MMP-3, MMP-13, Collagen II, and Aggrecan. (**K**) MMP-3/β-actin ratio. (**L**) MMP-13/β-actin ratio. (**M**) Collagen II /β-actin ratio. (**N**) Aggrecan/β-actin ratio. **P < 0.01

Correct Fig. 3:

**Fig. 3:**
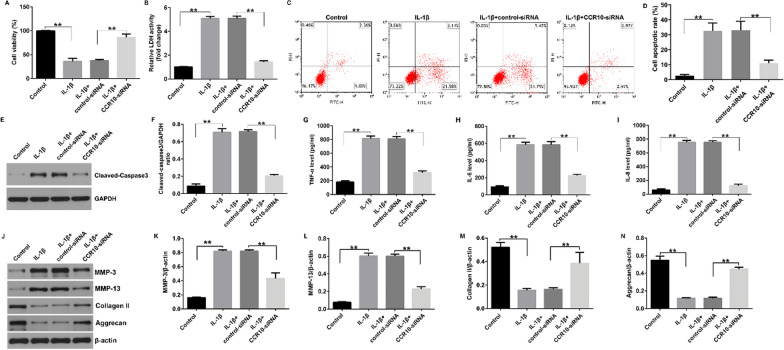
Effects of CCR10-siRNA on IL-1β-induced cell viability, apoptosis and inflammatory cytokines secretion. The CHON-001 cells were divided into four groups: control, IL-1β, IL-1β + control-siRNA, or IL-1β + CCR10-siRNA group. (**A**) Cell viability was assessed using MTT assay. (**B**) Analysis of LDH release. (**C**) Apoptosis was assessed by flow cytometry. (**D**) Quantification of apoptotic CHON-001 cells. (**E**) Western blot analysis of cleaved-caspase-3 expression. (**F**) Relatively cleaved-caspase-3 protein expression were quantified. The secretion of TNF-α (**G**), IL-6 (H) and IL-8 (**I**) were evaluated by ELISA. (**J**) Western blot analysis of MMP-3, MMP-13, Collagen II, and Aggrecan. (**K**) MMP-3/β-actin ratio. (**L**) MMP-13/β-actin ratio. (**M**) Collagen II /β-actin ratio. (**N**) Aggrecan/β-actin ratio. **P < 0.01

Incorrect Fig. 6:

**Fig. 6:**
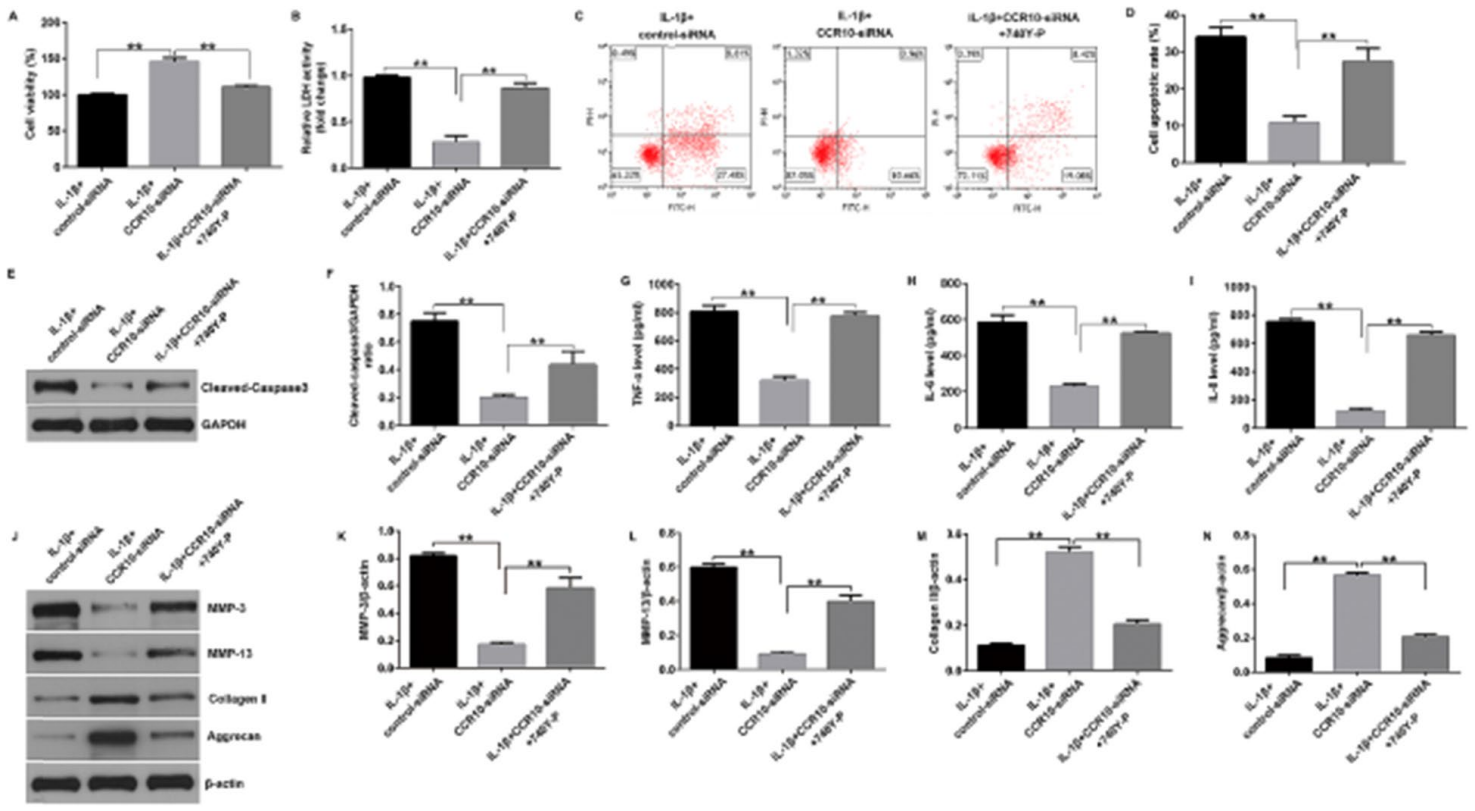
Effects of CCR10-siRNA or 740Y-P on IL-1β-induced cell viability, apoptosis, and inflammatory cytokines secretion. The CHON-001 cells were divided into three groups: IL-1β + control-siRNA, IL-1β + CCR10-siRNA, and IL-1β + CCR10-siRNA + 740Y-P groups. (**A**) MTT assay for cell viability. (**B**) Determination of LDH level. (**C**) Apoptotic cells were evaluated using flow cytometry. (**D**) Quantification of the apoptotic cells. (**E**) Determination of cleaved-caspase-3 expression. (**F**) Quantization of cleaved-caspase-3 expression. The levels of TNF-α (**G**), IL-6 (**H**), and IL-8 (**I**) were analyzed using ELISA. (**J**) Western blot analysis of MMP-3, MMP-13, Collagen II, and Aggrecan. (**K**) MMP-3/β-actin ratio. (**L**) MMP-13/β-actin ratio. (**M**) Collagen II /β-actin ratio. (**N**) Aggrecan/β-actin ratio. **P < 0.01

Correct Fig. 6:

**Fig. 6:**
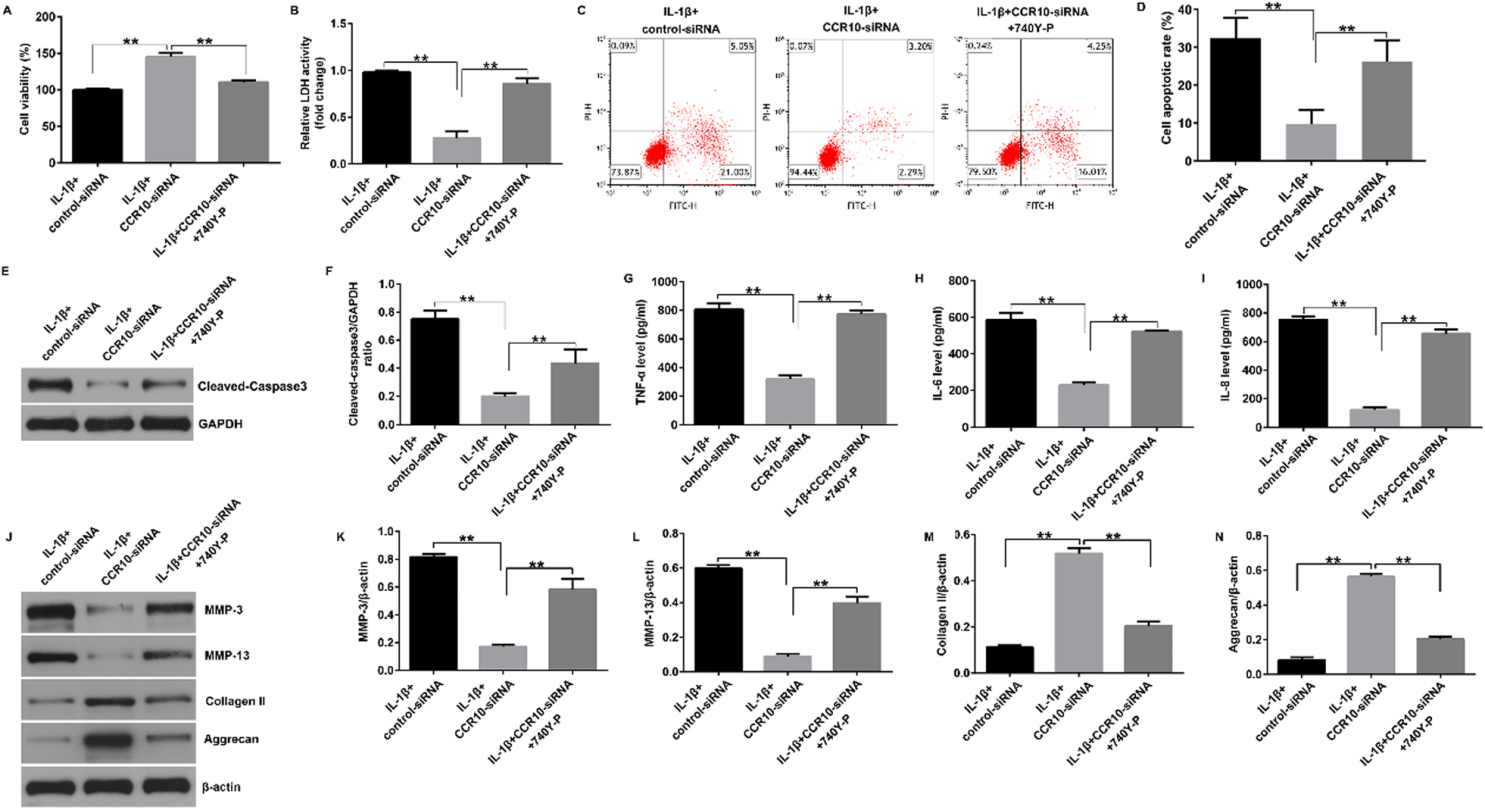
Effects of CCR10-siRNA or 740Y-P on IL-1β-induced cell viability, apoptosis, and inflammatory cytokines secretion. The CHON-001 cells were divided into three groups: IL-1β + control-siRNA, IL-1β + CCR10-siRNA, and IL-1β + CCR10-siRNA + 740Y-P groups. (**A**) MTT assay for cell viability. (**B**) Determination of LDH level. (**C**) Apoptotic cells were evaluated using flow cytometry. (**D**) Quantification of the apoptotic cells. (**E**) Determination of cleaved-caspase-3 expression. (**F**) Quantization of cleaved-caspase-3 expression. The levels of TNF-α (**G**), IL-6 (**H**), and IL-8 (**I**) were analyzed using ELISA. (**J**) Western blot analysis of MMP-3, MMP-13, Collagen II, and Aggrecan. (**K**) MMP-3/β-actin ratio. (**L**) MMP-13/β-actin ratio. (**M**) Collagen II /β-actin ratio. (**N**) Aggrecan/β-actin ratio. **P < 0.01

